# Supplementary material for: Protocol for a Delphi Consensus Study to Determine the Essential and Optional Ultrasound Skills for Medical Practitioners Working in District Hospitals in South Africa
Source: Int J Environ Res Public Health. 2022 Aug 5;19(15):9640. doi: 10.3390/ijerph19159640 (PMC9367781; doi:10.3390/ijerph19159640)
Supplement: Supplementary file 1 [file ijerph-19-09640-s001.zip › ijerph-1754305-Supplementary.pdf]

What ultrasound skills should a district level medical officer have?

Participant Questionnaire

I. Pre-amble

The following ultrasound skills have been taken from the American Academy of Family Physicians guideline document.

https://www.aafp.org/dam/AAFP/documents/medical_education_residency/program_directors/Reprint290D_POCUS.pdf

It has been simplified and divided into 10 clinical domains with specific subheadings for each specific ultrasound skillset. Please note that:

- All of these are to be considered for limited bedside ultrasound evaluation and not for comprehensive diagnostic ultrasound.
- Each skillset is to be graded as one of three possible options namely
  - 'Essential' – This skillset is an essential component to the required service delivery at a district level hospital and all medical officers should be able to perform this
  - 'Optional' – This skillset may be appropriate in some selected contexts, and can be seen as geographically specific, but it is not a necessary requirement for all medical officers at a district level hospital
  - 'Non-Essential' – This skillset is not appropriate for a district hospital and should not be performed at that level.
- There will be an opportunity for comment in each clinical domain, if there are clear themes in the comments this will be shared to all participants in following rounds.
- In the 1st round participants are encouraged to add any additional ultrasound skillset/s that they feel should also be considered. These will be included from the 2nd round.

1 OBS/GYN

1.1 First trimester evaluation:

1.1.1 Identification of the presence of an intrauterine pregnancy

1.1.2 Determine the viability of an intrauterine pregnancy

1.1.3 Detection of the fetal heart rate using M-Mode

1.1.4 First trimester pregnancy gestational age assessment by crown rump length detection

1.1.5 Recognition of molar pregnancy

1.2 Second/third trimester evaluation:

1.2.1 Determine placental position

1.2.2 Determine the fetal presentation

1.2.3 Perform a gestational age assessment and fetal weight estimation using abdominal circumference (AC), biparietal diameter (BPD), and femoral length (FL).

1.2.4 Assess the amniotic fluid volume using either the 4 quadrant calculation or deepest single pocket approach

1.2.5 Assess the placenta for features of placental abruption

1.2.6 Evaluation of cervical length to assess cervical insufficiency

1.2.7 Determine fetal gender after 18weeks

1.2.8 Assess fetal well-being using the biophysical profile

1.2.9 Assess fetal well-being during third trimester using umbilical artery doppler

1.2.10 Confirmation of fetal death

1.3 Gynecology ultrasound skills

1.3.1 Confirmation of intrauterine device (IUD) position

1.3.2 Measurement of endometrial thickness

1.3.3 Assessment of an adnexal mass: simple, complex and haemorrhagic cysts

1.3.4 Identification of an ovarian torsion

1.3.5 Assessment of a breast mass

2 Cardiac

2.1 Detection of a pericardial effusion using the sub-xiphoid view

2.2 Assessment of global left ventricle contractility (hyperdynamic / normal / decreased)

2.3 Calculation of left ventricle ejection fraction through radial contractility (Teicholtz)/ mitral annular plane systolic excursion (MAPSE)

2.4 Assessment of left ventricular hypertrophy

2.5 Assessment of diastolic dysfunction

2.6 Assessment of regional wall motion abnormalities

2.7 Assessment of valvular abnormalities

2.8 Assessment of right ventricle size and strain and the possibility of PE in the appropriate clinical setting

2.9 Assessment of right ventricle function using tricuspid annular plane systolic excursion (TAPSE)

2.10 Measurement of inferior vena cava (IVC) diameter and collapsibility to approximate volume status

3 Trauma

3.1 Assessment of free fluid in the abdominal cavity

3.2 Assessment of free fluid around the heart with a subxyphoid view

3.3 Assessment of a pneumothorax

3.4 Assessment of a haemothorax

4 Abdominal

4.1 Aorta

4.1.1 Detection of an abdominal aortic aneurysm

4.1.2 Detection of an abdominal aortic dissection

4.1.3 Detection of a proximal aortic root aneurysm or dissection

4.2 Hepatobilliary

4.2.1 Assess the gallbladder for cholelithiasis

4.2.2 Assess for acute cholecystitis

4.2.3 Assess for common bile duct (CBD) obstruction (Choledocholithiasis)

4.2.4 Assess for hepato-splenomegaly

4.3 Bowel

4.3.1 Assess for appendicitis

4.3.2 Identify an abdominal wall hernia

4.3.3 Identify an inguinal hernia

4.4 Genitourinary tract

4.4.1 Assess renal size

4.4.2 Identify and grade hydronephrosis

4.4.3 Identify urinary retention and post-void residual volume

4.4.4 Identify a varicocele

4.4.5 Identify a testicular torsion

4.4.6 Identify epididymo-orchitis

4.4.7 Asses prostate volume

5 Vascular

5.1 Identify lower extremity deep vein thrombosis (DVT) in low risk cases with 2-Zone discrimination technique

5.2 Identify lower extremity DVT with colour Doppler, and graded compression of the entire limb

5.3 Identify upper extremity DVT with colour Doppler, and graded compression of the entire limb

6 Soft tissue and musculoskeletal

6.1 Differentiating cellulitis versus abscess in soft tissue
6.2 Identification of a foreign body
6.3 Identification of a joint effusions/bursitis
6.4 Identification of long bone fractures
6.5 Identification of tendon rupture/tear
6.6 Identification of tendonitis/tendinopathy
6.7 Identification of rotator cuff injury
6.8 Identification of a ligament injury of the knee
6.9 Identification of a ligament injury of the ankle
6.10 Assessment of carpal tunnel syndrome

7 Pulmonary

7.1 Identification of pulmonary oedema
7.2 Identification of a lobar pneumonia
7.3 Identification of pneumothorax
7.4 Identification of pleural effusion or hemothorax
7.5 Assessment of pleural mass
7.6 Identification of interstitial pneumonia
7.7 Identification of Acute Respiratory Distress Syndrome (ARDS)
7.8 Identification of chronic interstitial lung disease

8 Ocular

8.1 Identification of detached retina
8.2 Identification of vitreous detachment
8.3 Identification of vitreous haemorrhage
8.4 Identification of intraocular foreign body
8.5 Measurement of posterior Ocular Nerve Sheath Diameter for assessment of intracranial pressure (papilledema)
8.6 Identification of a lens dislocation

9 Ultrasound use for procedural guidance in

9.1 Thoracentesis
9.2 Paracentesis
9.3 Peripheral IV placement
9.4 Central line placement
9.5 Lumbar puncture
9.6 Knee aspiration and injection
9.7 Foreign body identification and removal
9.8 Fine needle aspiration/biopsy
9.9 Shoulder, ankle, hip, wrist aspiration, and injection
9.10 Peripheral nerve blocks

10 Ultrasound based clinical protocols

10.1 FAST/E-FAST: Focused assessment with sonography for trauma

10.2 RUSH: Rapid ultrasound for shock and hypotension

10.3 BLUE: Bedside lung ultrasound in emergency

10.4 CLUE: Cardiac limited ultrasound exam

10.5 FEEDS (Fundal, Eccentric, Elliptical, Decidual Reaction, Size of Gestational sac) criteria: To determine presence IUP
